# Multifactorial Refractory Acne in Women: Insights from a Case Series Involving Hormonal-, Metabolic-, and Corticosteroid-Related Triggers

**DOI:** 10.3390/life15081196

**Published:** 2025-07-28

**Authors:** Alexa Florina Bungau, Ruxandra Cristina Marin, Delia Mirela Tit, Gabriela Bungau, Ada Radu, Daciana Elena Branisteanu, Laura Maria Endres

**Affiliations:** 1 Doctoral School of Biomedical Sciences, Faculty of Medicine and Pharmacy, University of Oradea, 410087 Oradea, Romania; prada.alexaflorina@student.uoradea.ro (A.F.B.); gbungau@uoradea.ro (G.B.); adaradu@uoradea.ro (A.R.); lendres@uoradea.ro (L.M.E.); 2 Department of Preclinical Disciplines, Faculty of Medicine and Pharmacy, University of Oradea, 410073 Oradea, Romania; 3 Department of Pharmacy, Faculty of Medicine and Pharmacy, University of Oradea, 410028 Oradea, Romania; 4 Discipline of Dermatology, Faculty of Medicine, Grigore T. Popa University of Medicine and Pharmacy, 700115 Iasi, Romania; daciana.branisteanu@umfiasi.ro; 5 Department of Psycho-Neurosciences and Recovery, Faculty of Medicine and Pharmacy, University of Oradea, 410073 Oradea, Romania

**Keywords:** acne vulgaris, treatment-resistant acne, adult-onset acne, polycystic ovary syndrome, metabolic syndrome, corticosteroid-induced acne, multidisciplinary approach, dermatologic therapy

## Abstract

Acne vulgaris is a multifactorial inflammatory skin disorder that significantly impairs quality of life and may signal underlying systemic dysfunction, particularly in adult women with treatment-resistant or atypical presentations. This case series presents three clinically and etiologically distinct examples of persistent acne in female patients, each associated with different contributing factors: long-term topical corticosteroid misuse, polycystic ovary syndrome (PCOS), and metabolic syndrome with autoimmune thyroiditis. All cases underwent comprehensive dermatologic evaluation, endocrine/metabolic assessments, and personalized therapeutic interventions, ranging from corticosteroid withdrawal and barrier repair to hormonal modulation and insulin-sensitizing therapy. Clinical progression was monitored for up to six months, revealing favorable responses in all cases, with substantial lesion clearance and improved skin quality. These real-world cases highlight the importance of an integrative, interdisciplinary diagnostic approach in refractory acne and support the need for individualized, long-term management strategies tailored to underlying systemic contributors.

## 1. Introduction

Acne vulgaris is one of the most common dermatological conditions, affecting individuals across different age groups and socioeconomic backgrounds. Globally, it ranks as the eighth most frequent skin disease, with an age-standardized prevalence of approximately 9.4%, affecting an estimated 231 million individuals in 2020 and accounting for 117 million new cases annually [1].

The overall disease burden, measured in disability-adjusted life years, has increased by nearly 48% since 1990. Adolescents are disproportionately impacted, with global incidence estimates exceeding 85% and around 20% having moderate to severe acne. Adult-onset acne is also increasingly recognized, affecting up to 40% of the adult population [2].

The burden of acne shows notable geographic and demographic variability. Europe has some of the highest rates of disease. Population-based surveys in Europe report overall prevalence rates of 5.4% in the general population. According to 2021 data, Western Europe has the greatest age-standardized frequency internationally, with acne being almost 25% more common in young women (10,912 per 100,000) than in young males (8728 per 100,000). Age remains the most significant epidemiological determinant: adolescents aged 15–19 exhibit the highest incidence, and those aged 10–14 have shown the largest annual percentage increase over the past three decades [3]. Acne affects around 1.5% of those over the age of 25 and 3.9% of those aged 25 and under, according to 2020 data [4].

Despite its high distribution, acne vulgaris has a negative and psychosomatically significant impact on the quality of life of people affected, particularly those with chronic or severe illness. Acne has been strongly associated with anxiety and depression, underscoring the need for early and effective intervention. A meta-analysis of 42 studies found a significant association between acne and both depression and anxiety (*p* < 0.0001) [5]. Similarly, a 2023 community-based study conducted by Morshed et al. demonstrated strong correlations between acne severity and markers of depression, anxiety, and stress (for all *p* < 0.001), confirming that acne substantially impairs mental well-being [6].

Given the context above, a comprehensive understanding of acne’s pathophysiology and clinical manifestations is essential. The pathogenesis of acne vulgaris is complex and multifactorial, involving hormonal, microbial, immunological, and environmental influences. At its core, acne is a chronic inflammatory disorder of the pilosebaceous unit, driven by a complex interplay of follicular hyper-keratinization, androgen-induced sebum overproduction, pilosebaceous duct obstruction, and a resulting inflammatory cascade. Thus, four primary pathogenic pathways have been identified: hormonally induced hyper-seborrhea, follicular hyper-keratinization, *Cutibacterium acnes* (*C. acnes*) colonization of the pilosebaceous unit, and the subsequent local inflammatory response [7,8].

Clinically, it appears as a variety of lesions ranging from non-inflammatory comedones to inflammatory papules, pustules, and, in more severe cases, nodules and cysts, typically distributed across the face, chest, and back. Although it most commonly arises during adolescence, acne may persist into adulthood or even develop de novo, with adult women being disproportionately affected. Studies demonstrate that approximately half of women in their 20s and over a quarter of those in their 40s suffer from active acne, challenging the misconception that this condition exclusively affects adolescents [9].

Hormonal imbalances, particularly androgen excess, play a key role in female acne, as seen in conditions such as polycystic ovary syndrome (PCOS), where sebum production and follicular keratinization are markedly increased [10].

In recent years, acne vulgaris has been increasingly linked to metabolic syndrome, with mechanisms implicating insulin resistance, dyslipidemia, and low-grade systemic inflammation. Okoro et al. and Fatima et al. indicated that hyperinsulinemia promotes ovarian and adrenal androgen synthesis while reducing hepatic production of sex hormone-binding globulin (SHBG), thereby increasing the levels of bioavailable androgens that stimulate sebaceous gland activity [11,12]. In parallel, elevated insulin-like growth factor 1 (IGF-1) levels stimulate sebocyte proliferation and lipogenesis, enhancing comedogenesis and inflammation [13,14].

Dyslipidemia may also alter sebum composition, promoting a pro-inflammatory environment in the pilosebaceous unit. Additionally, systemic low-grade inflammation and oxidative stress commonly observed in metabolic syndrome amplify local cytokine activity (e.g., TNF-α, IL-1β), further exacerbating acne severity, as it was shown in a study by Chandak et al. [15]. These pathways converge to create a cutaneous microenvironment that favors lesion persistence, particularly in adult women with metabolic abnormalities, as seen in clinical and translational studies. Clinical observations by Bungau et al. showed that features of metabolic syndrome, such as obesity, insulin resistance, and dyslipidemia, create a hormonal and inflammatory milieu that perpetuates acne lesions [16]. These conditions are further linked to elevated insulin-like growth factor 1 (IGF-1), which enhances sebaceous gland activity and promotes comedogenesis [15].

The microenvironment of the pilosebaceous unit plays a crucial role in lesion development. Follicular hyper-keratinization leads to ductal obstruction and sebum retention, providing a substrate for *C. acnes* proliferation. This bacterium contributes to inflammation by producing pro-inflammatory free fatty acids and biofilms, which stimulate innate immunity through toll-like receptor (TLR)-2 and -4. This process triggers the release of cytokines, such as interleukin (IL)-1α and -8, tumor necrosis factor (TNF)-α, and leukotriene B4 (LTB_4_) [17,18].

Inflammatory amplification is sustained by oxidative stress, nuclear factor kappa B (NF-κB) activation, and the upregulation of antimicrobial peptides, like cathelicidins and β-defensins. Microbial dysbiosis, especially involving virulent strains of *C. acnes* and *Staphylococcus epidermidis*, further aggravates local immune responses [19]. Pharmacological factors can also influence acne severity. Corticosteroid therapy, whether systemic or topical, has been associated with acneiform eruptions and may worsen existing lesions by altering follicular keratinization and local immune responses [20].

Considering the multifactorial pathogenesis of acne vulgaris and the growing body of evidence linking it to hormonal and metabolic imbalances, identifying contributing factors in patients with treatment-resistant disease is essential. This is particularly relevant in adult female patients, where endocrine or metabolic abnormalities may underlie persistent acne despite conventional management. This case series explores the diagnostic and therapeutic complexity of refractory acne in adult female patients by presenting three representative cases associated with distinct systemic comorbidities. The objective is to emphasize the importance of interdisciplinary evaluation and individualized treatment approaches in the management of persistent or atypical acne.

## 2. Materials and Methods

This case series presents three consecutively selected cases of treatment-resistant acne, identified during dermatological consultations at an outpatient medical center in Oradea, Romania, between 2023 and 2024. All patients underwent comprehensive clinical and paraclinical evaluations. In each case, a detailed medical history was recorded, followed by a complete dermatological examination. Where clinically indicated, additional investigations were performed, including hormonal and metabolic assessments and pelvic ultrasonography. Acne severity was classified using the well-known six-grade Global Evaluation Acne (GEA) scale, which ranges from “clear” (grade 0) to “very severe” (grade 5). The scale incorporates the number, types, and distribution of lesions, with clear definitions [21,22]. GEA grades were used consistently at baseline and follow-up to support objective evaluation of therapeutic response across cases.

Eligible patients were adult women presenting with moderate-to-severe, treatment-resistant acne that warranted interdisciplinary evaluation. Additional inclusion criteria included the availability of comprehensive dermatological and paraclinical records, including hormonal and metabolic assessments, as well as informed consent for clinical monitoring and the publication of anonymized data. The selected cases illustrate distinct clinical forms of acne, as follows: corticosteroid-induced acne, acne associated with PCOS, and adult-onset acne in metabolic syndrome.

Therapeutic interventions were individualized according to the suspected etiology and included systemic treatments (antibiotics, oral contraceptives, and metformin), targeted topical regimens, and adjunctive measures, such as cosmetic procedures and lifestyle modifications.

Clinical progression was monitored through scheduled follow-up visits, according to clinical need, typically occurring around 2 to 6 months after treatment initiation. In selected cases, earlier evaluations were performed to monitor acute changes, such as rebound flares. As part of their ongoing care, patients were encouraged to document their skin evolution using personal smartphone photographs, taken under similar lighting and angle conditions where possible. These images were reviewed by the attending dermatologist to support therapeutic evaluation. All photographs included in this manuscript were provided voluntarily by the patients and form part of the personal archive of the last author, who was directly involved in their clinical management.

All the ethical requirements regarding patients were respected, and the informed consent that was obtained from all subjects involved in this study allowed using their data in future publications. This study was conducted in accordance with the Declaration of Helsinki and approved by the Ethics Committee for Scientific Research of the Pelican Hospital, Oradea, Romania (Decision 41/9 January 2023).

## 3. Case Presentation

We will present the three investigated cases below, both in detail and comparatively, for a better understanding.

### 3.1. Case 1

**Figure 1:**
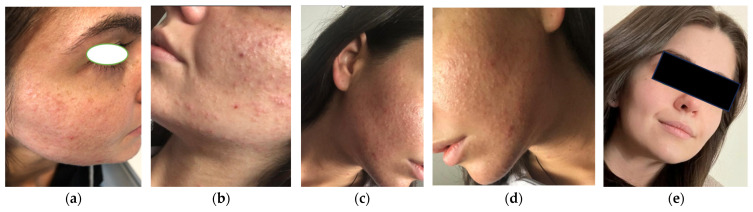
Initial presentation of steroid-induced acne vulgaris flare-up disseminated with closed and open comedones on the face, inflammatory papules, hyperpigmentation, erythema, atrophic scars, hyper-seborrhea, post-traumatic crusts and lesions, telangiectasia, local hypersensitivity, and mild scaling (**a**,**b**). Ten-day follow-up (**c**,**d**). Post-treatment outcome after six months of combined systemic and topical therapy: the lesions had completely resolved (**e**).

A 21-year-old female patient from a rural environment, with no significant medical history, presented with persistent, polymorphic facial lesions. Clinical examination revealed both open and closed comedones on the face, numerous inflammatory papules, diffuse erythema, post-inflammatory hyperpigmentation, punctate atrophic scars secondary to prior lesions, and localized telangiectasias (Figure 1a,b). The skin appeared seborrheic and shiny, with visible sebum excess, along with fine adherent scales and serous crusts at sites of excoriation. The patient reported intense pruritus, a burning sensation, and notable facial discomfort, symptoms that are atypical for classical acne vulgaris.

Detailed history-taking revealed a critical contributing factor. The patient had been applying a topical corticosteroid cream (methylprednisolone aceponate 0.1%) to her face since the age of 7, in the absence of medical follow-up. The long-term, unsupervised use of facial corticosteroids had no connection to dermatologic care provided at our center. This product had been used frequently (daily or every other day) to manage recurrent erythematous eruptions. The long-term use of topical corticosteroids significantly altered the clinical presentation of her acne. While initially suppressing inflammation, the chronic corticosteroid application ultimately contributed to the development of corticosteroid-induced acneiform lesions and telangiectasias, thereby complicating the diagnosis and course of the disease.

Although demodicosis was considered as a differential diagnosis, given the chronic corticosteroid use, the absence of classical features (like follicular scaling, centro-facial papulopustules, or ocular involvement) and the overall clinical morphology and lesion distribution were not consistent with demodicosis and favored an acneiform eruption.

The established diagnosis was moderate acne vulgaris (GEA grade 3) induced by prolonged topical corticosteroid use, a condition often referred to as cortico-dependent acne, characterized by rebound flares upon discontinuation of treatment.

Systemic therapy was initiated with doxycycline 100 mg daily for three months, along with an oral antihistamine (desloratadine) to alleviate pruritus. Discontinuation of the corticosteroid was strongly advised after 14 years of unsupervised use. To support skin barrier recovery and manage withdrawal-related flare-ups, a comprehensive dermato-cosmetic regimen was introduced, including micellar cleansing water and barrier-repair moisturizers designed for sensitive, acne-prone skin. Following the withdrawal of topical corticosteroids, a temporary flare-up of the eruption was observed, consistent with a rebound phenomenon. Within the first 1–2 weeks, the patient developed new erythematous papules and inflammatory pustules. Despite this, continued systemic and topical therapy led to gradual regression of the lesions. At the 10-day follow-up (Figure 1c,d), both erythema and the burning sensation had improved, while the typical features of acne vulgaris (comedones, papules, and pustules) became more prominent, indicating that the suppressive effects of corticosteroids had dissipated. To consolidate therapeutic outcomes, a structured, multimodal treatment plan was maintained, as outlined in Table 1. In addition, the patient underwent monthly manual comedone extraction, performed under sterile conditions, as an adjunctive measure to accelerate the resolution of retention lesions and support clinical recovery.

Under this regimen, the patient achieved a favorable response. After approximately six months, inflammatory lesions had completely resolved, with no new outbreaks observed. Residual findings included only mild atrophic scarring and post-inflammatory hyperpigmented areas. The acne severity had decreased to GEA grade 1, reflecting near-complete remission with minimal comedonal activity and no active inflammation. A long-term maintenance strategy was implemented, including non-comedogenic skincare and intermittent topical retinoids or fruit acids to prevent recurrence. At the latest follow-up (Figure 1e), the skin appeared stable, with no signs of flare-up under continuous dermatologic supervision.

### 3.2. Case 2

**Figure 2:**
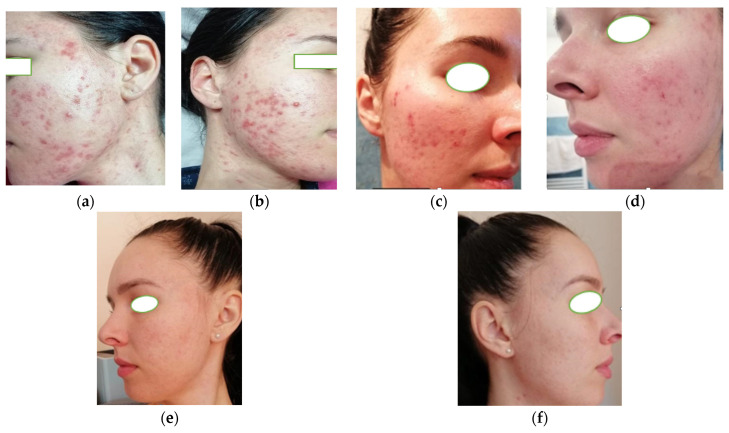
Multiple inflammatory papules on the face and upper part of the neck (**a**,**b**); multiple closed and open comedones, accentuated seborrhea, and multiple atrophic scars on the cheeks, chin, forehead, and upper part of the neck. Favorable results at 3 months (**c**,**d**). At 6 months, fine post-acne scars and complete remission of acne (**e**,**f**).

A 26-year-old female patient from a rural area presented with chronic, treatment-resistant acne. On clinical examination, she exhibited numerous inflammatory papules on the face, including the mandibular region and cheeks, as well as on the upper neck. A significant number of both open and closed comedones were observed, accompanied by pronounced seborrhea. Notably, the facial skin displayed multiple atrophic scars distributed across the cheeks, chin, and forehead, indicating a history of severe acne flare-ups (Figure 2a,b). Prior to presentation, the patient had undergone conventional acne treatments over the course of approximately 9 to 12 months, including multiple regimens of topical antibiotics (clindamycin, erythromycin), retinoids (adapalene, tretinoin), and a short oral doxycycline course (6 weeks). Despite consistent adherence, these interventions failed to achieve lasting remission, with persistent inflammatory lesions and recurrent flares. In the conditions of this therapeutic resistance, a broader diagnostic approach was pursued, including endocrinological and gynecological assessment. Endocrinological and gynecological evaluations revealed polycystic ovary syndrome (PCOS): pelvic ultrasound demonstrated micro-polycystic ovaries, and hormonal profiling indicated mild hyperandrogenism. Based on these findings, the patient was prescribed hormonal therapy consisting of combined oral contraceptives (ethinyl estradiol and cyproterone acetate) to manage PCOS and correct the underlying hormonal imbalance. The recommended duration of therapy was at least six months.

The concurrent dermatological diagnosis was moderate-to-severe persistent acne vulgaris (GEA grade 4) associated with PCOS.

Systemic dermatologic treatment was initiated with doxycycline 100 mg daily, in parallel with the introduction of a combined oral contraceptive, as recommended by the gynecologist. A comprehensive skincare and pharmacological regimen was implemented, as summarized in Table 2.

In addition to pharmacologic therapy, the patient underwent monthly adjunctive cosmetic procedures, including comedone extraction and microneedling (derma pen) sessions aimed at improving post-acne atrophic scarring. Treatment adherence was excellent, with the patient following both the dermatological protocol and the hormonal regimen as prescribed.

At the 3-month follow-up, there was a marked reduction in the number of inflammatory lesions and a noticeable decrease in sebaceous secretion. Atrophic scars appeared less prominent, largely due to the combined effects of microneedling and retinoid-induced dermal remodeling (Figure 2c,d). Clinically, the acne severity had improved to GEA grade 2, reflecting mild persistent comedonal activity with minimal inflammation.

By the 6-month mark, the acne was nearly completely resolved, with only occasional mild lesions noted premenstrually. At this point, the disease severity corresponded to GEA grade 1, reflecting near-complete remission with minimal inflammatory activity (Figure 2e,f). Additionally, the patient reported improved menstrual cycle regularity under PCOS-targeted hormonal therapy.

Despite the favorable therapeutic response, it is noteworthy that following discontinuation of oral contraceptives (after the recommended course), the patient experienced a relapse of acne within a few months. This recurrence coincided with worsening of her metabolic profile; subsequent testing revealed elevated cholesterol and triglyceride levels. This evolution highlights the importance of continuous monitoring and the need for a long-term maintenance strategy. In such cases, resumption of hormonal therapy or initiation of alternative systemic interventions (such as metformin or targeted dietary management) may be necessary to prevent recurrence of acne in the context of PCOS and emerging metabolic dysfunction.

### 3.3. Case 3

A 40-year-old female patient, the mother of two children, presented with late-onset acne that had developed approximately one year prior. She reported no personal history of acne during adolescence or early adulthood. Medical history revealed a lifelong tendency toward seborrheic skin, but without any prior acneiform eruptions until the current episode.

**Figure 3:**
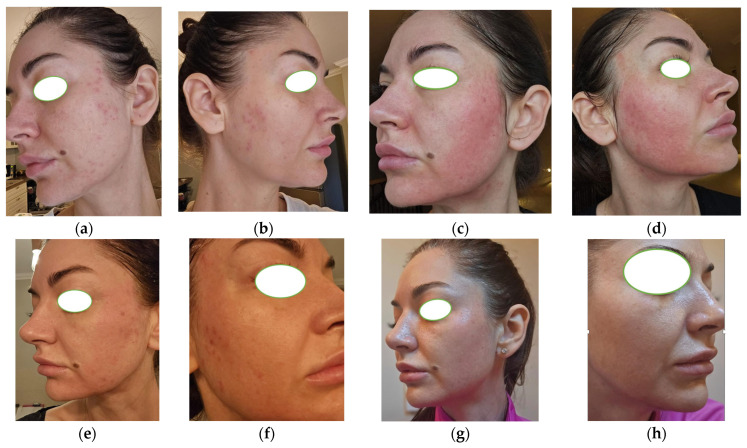
Initial presentation of late-onset acne in a 40-year-old woman: painful inflammatory papules, multiple closed and open comedones, and facial erythema (**a**,**b**). After 3 weeks: facial erythema (**c**,**d**). After 2 months, fine post-acne scars, multiple closed and open comedones, and facial erythema (**e**,**f**). At 6 months, complete remission of acne (**g**,**h**).

The current lesions began around 12 months prior as a few comedones and pustules, which gradually progressed in both number and severity. Clinical examination revealed multiple erythematous inflammatory papules, some of which were tender to palpation, as well as numerous open and closed comedones distributed across the face. The lesions were predominantly localized to the U-zone (cheeks and chin), a distribution pattern characteristic of adult female acne. Diffuse facial erythema was noted, especially over the malar and mental regions, along with persistent erythematous-telangiectatic areas, particularly on the nasal alae. There were no nodules or deep cystic lesions observed, and scarring was absent, consistent with the relatively recent onset of the condition. Given the late-onset presentation, an interdisciplinary investigation was initiated to explore potential underlying etiologies (Figure 3a,b).

Given the patient’s age and atypical presentation, further systemic evaluation was initiated. Prior to presentation, the patient had undergone a standard course of topical acne therapy, including benzoyl peroxide and a fixed combination of clindamycin with adapalene, over a period of 12 weeks, with only partial and transient improvement. Further investigations revealed a euthyroid hormonal profile; however, positive antithyroid antibodies confirmed the presence of autoimmune Hashimoto’s thyroiditis, currently without biochemical dysfunction. Routine laboratory tests identified mixed dyslipidemia, moderately elevated total and LDL cholesterol, mildly increased triglycerides, and impaired fasting glucose (105 mg/dL), suggesting early-stage metabolic syndrome. The patient’s body mass index (BMI) was 29 kg/m^2^, consistent with overweight, bordering on class I obesity. Abdominal circumference exceeded 88 cm, and blood pressure was at the upper limit of normal (130/85 mm Hg). Taken together, these findings confirmed the presence of the metabolic syndrome characterized by abdominal obesity, dyslipidemia, and impaired glucose tolerance. A diabetology consultation was also recommended.

The dermatological diagnosis was late-onset acne vulgaris (GEA grade 3) in the context of underlying metabolic disturbances. The coexisting euthyroid autoimmune thyroiditis was considered likely incidental, with no direct causal link to the acne. A multidisciplinary management plan was instituted, as detailed in Table 3.

Given the patient’s metabolic profile, hormonal contraceptives were not prescribed (menstrual cycles were regular, and there were no clinical signs of hyperandrogenism). Instead, metabolic optimization was prioritized. After three weeks of combined dermatologic and metabolic intervention, inflammatory lesions showed early improvement (Figure 3c,d).

After approximately two months, the patient reported a modest reduction in inflammatory lesions and decreased seborrhea (Figure 3e,f), along with a 3 kg weight loss and normalization of fasting glucose. Clinically, the acne severity remained at GEA grade 3, consistent with moderate acne, despite partial improvement. At the six-month follow-up, over 70% of the active acne lesions had resolved, with no significant new eruptions. The residual severity was graded as GEA grade 2, indicating mild comedonal and occasional inflammatory activity under stable dermatologic control (Figure 3g,h).

The patient demonstrated sustained clinical improvement. Although the facial skin remained seborrheic, the number of open and closed comedones was markedly reduced compared to baseline. Inflammatory lesions were minimal, and no new significant eruptions had occurred. The patient continued adherence to topical maintenance therapy and underwent regular cosmetic procedures, including comedone extraction, to support lesion control and prevent recurrence. This case illustrates a form of adult-onset acne associated with systemic factors, demonstrating a satisfactory clinical response to an integrated treatment approach targeting both cutaneous and metabolic components.

For comparison purposes, Table 4 summarizes the most relevant clinical, therapeutic, and response-related parameters across all three patients. By aligning key features, such as acne phenotype, GEA severity grades, systemic associations, and treatment outcomes, this comparative overview illustrates both the heterogeneity in acne presentations in women and the effectiveness of individualized, interdisciplinary interventions.

## 4. Discussion

Through three distinct clinical scenarios, this case series highlights how persistent acne may signal underlying systemic imbalances that complicate both diagnosis and management. Our goal was to reflect real-world clinical decisions in patients where standard dermatologic approaches, such as topical retinoids, benzoyl peroxide, and systemic antibiotics, had already been tried but proved insufficient (as in Cases 2 and 3), or where the clinical picture had been significantly altered by long-term, unsupervised corticosteroid use (as in Case 1). These conventional therapeutic strategies were employed in accordance with European acne management guidelines, specifically those issued by the European Dermatology Forum (EDF) and the Global Alliance to Improve Outcomes in Acne, which remain the benchmark for evidence-based care in this field [23,24].

Although oral isotretinoin is widely regarded as the treatment of choice for severe or refractory forms of acne, particularly nodulocystic variants, its use is not without limitations. Isotretinoin targets all major pathogenic pathways of acne: follicular hyper-keratinization, sebaceous gland hyperactivity, *Cutibacterium acnes* proliferation, and inflammation [25]. However, it requires close monitoring of hepatic enzymes, lipid profiles, and neuropsychiatric symptoms, with common side effects including xerosis, cheilitis, epistaxis, and musculoskeletal discomfort [26]. Moreover, due to its teratogenicity (FDA category X), strict contraceptive measures and pre-treatment counseling are mandatory for women of reproductive age [27]. While isotretinoin remains a cornerstone in acne management, the patients presented in this series either declined its use or were not ideal candidates due to comorbidities or personal preferences. These examples illustrate that comparable clinical improvement can be achieved through alternative, individualized, and interdisciplinary treatment strategies that prioritize patient safety, adherence, and quality of life.

Moreover, in all three cases, acne was associated with systemic abnormalities extending beyond the dermatologic domain, including PCOS, autoimmune thyroiditis, dyslipidemia, impaired glucose metabolism, or obesity, conditions frequently associated with systemic inflammation, hormonal dysregulation, and overweight status. All these may contribute to acne persistence or atypical presentation. Consequently, when standard acne treatments fail or clinical patterns deviate from classical forms, a thorough reassessment of underlying systemic factors becomes essential. Interdisciplinary evaluation may not only clarify etiology but also inform more effective, long-term therapeutic strategies.

The cases emphasize the significant influence of endocrine and metabolic disorders on acne evolution, particularly in young and adult female patients. In Case 2 and Case 3, the patients exemplify two distinct yet converging pathogenic pathways. In the first, ovarian androgen excess due to PCOS sustained acne, while in the latter, insulin resistance and dyslipidemia (components of metabolic syndrome) created a pro-inflammatory and potentially hyperandrogenic environment. Hormonal imbalances, such as those seen in PCOS, further complicate acne management. Hyperandrogenism stimulates sebaceous gland hyperplasia and sebum production, which promotes *Cutibacterium acnes* colonization and inflammation of the pilosebaceous follicles, mechanisms well established in acne pathophysiology. Insulin resistance, a key feature of metabolic syndrome, results in compensatory hyperinsulinemia, increased IGF-1 secretion, and reduced levels of sex hormone-binding globulin (SHBG), thereby increasing free androgen bioavailability and amplifying inflammatory pathways [28]. Recent studies have reported a higher prevalence of metabolic syndrome among individuals with severe acne compared to the general population, suggesting a bidirectional relationship between these conditions [16]. Furthermore, increased oxidative stress has been observed in patients with acne associated with metabolic disturbances, indicating a self-perpetuating inflammatory cycle that may require targeted interventions beyond conventional dermatologic therapies [13].

The first case illustrates a distinct acne pathogenesis triggered by prolonged topical corticosteroid use, an increasingly recognized cause of acneiform eruptions. Corticosteroids can disrupt follicular keratinization and suppress local immunity, leading to papulopustular lesions often mistaken for classical acne, as it was shown by Saraswat et al. and Maskey et al. [29,30]. Sudden withdrawal following chronic use may precipitate a rebound flare characterized by intense inflammation, as previously reported by Harlan in steroid withdrawal syndromes [31]. This rebound phenomenon requires careful pharmacologic management, typically involving gradual steroid withdrawal, systemic anti-inflammatory support, and tailored reintroduction of standard acne therapies.

The patient’s long-term, unsupervised application of methylprednisolone aceponate from early childhood contributed to the development of steroid-modified acne. Pediatric skin exhibits increased permeability, making children particularly susceptible to corticosteroid side effects. Sharma et al. emphasized the dangers of facial corticosteroid misuse, especially among younger patients, highlighting a pattern of delayed diagnosis and rebound flares [32]. Mahar et al. reported that 38% of facial steroid users developed acneiform eruptions, with 19% exhibiting persistent erythema or telangiectasias [33]. Similarly, Jain et al. found that 45% of patients with a history of facial steroid misuse developed steroid-induced acne, often with atypical features such as post-inflammatory hyperpigmentation and dermal atrophy [34]. Corticosteroids enhance follicular hyper-keratinization and alter sebaceous gland morphology, promoting comedogenesis and inflammation. Histological findings included epidermal thinning, sebaceous gland hyperplasia, and perivascular inflammatory infiltrates, features consistent with our patient’s clinical presentation [35].

To control the inflammatory rebound and allow skin barrier restoration, doxycycline was selected for its dual antimicrobial and anti-inflammatory activity [36], especially critical during the acute flare phase, as Kim et al. indicated in their research [17]. This was followed by the introduction of a comprehensive topical regimen including benzoyl peroxide, clindamycin, erythromycin, and azelaic acid, agents that collectively address microbial overgrowth, follicular obstruction, and pigmentation abnormalities [37,38]. These combinations are validated by international guidelines as effective multi-target therapies for acne and resistance prevention [39]. In a study by Kurokawa et al., barrier repair and skin comfort were prioritized using ceramide-based moisturizers and mild, pH-balanced cleansers [40]. This supportive care facilitated adherence and reduced irritation, which is especially relevant in patients with prior skin barrier compromise. Previous studies emphasized the importance of adjunctive dermo-cosmetics in optimizing therapeutic response and minimizing relapses in patients with post-inflammatory dyschromia and steroid-modified skin. An 8-week, split-face, double-blinded randomized controlled trial, including 40 patients with mild-to-moderate acne, by Tempark et al. found that a ceramide + niacinamide-containing moisturizer (CCM), when used alongside benzoyl peroxide and adapalene, significantly improved both inflammatory and non-inflammatory acne lesions, reduced skin irritation, and restored barrier function compared to a hydrophilic cream [41].

Such skincare supports therapeutic adherence, minimizes relapses, and is especially valuable in steroid-modified skin with compromised barrier integrity. Shibata et al. showed that glucocorticoids markedly enhanced TLR2 expression in keratinocytes, amplifying *C. acnes*-induced inflammatory signaling, which suggests a plausible mechanism for the exaggerated poststeroid flare in our patient [42]. These findings further support the need for transitional anti-inflammatory strategies, such as systemic antibiotics, during the withdrawal period. The therapeutic approach taken in this case aligned with both American and European acne treatment guidelines, which recommend systemic antibiotics like doxycycline as first-line agents for moderate-to-severe inflammatory acne, especially in cases complicated by comorbid mechanisms or resistance to topicals [23,24,43]. This case demonstrates the clinical complexity of corticosteroid-induced acne and highlights the need for multidisciplinary care. Dermatologic intervention, skin barrier restoration, patient education, and responsible prescribing practices are essential to prevent chronic steroid misuse and its dermatologic consequences.

In Case 2, the combination of systemic doxycycline and a combined oral contraceptive (COC) reflects a widely accepted therapeutic strategy for managing acne associated with PCOS. COCs mitigate androgenic stimulation of sebaceous glands and have been shown to significantly reduce acne severity, particularly when used in conjunction with systemic antibiotics during the initial treatment phase [44,45,46]. This therapeutic approach contributed substantially to acne remission in our patient, confirming the importance of addressing systemic drivers of disease. However, the recurrence of lesions following COC discontinuation and the onset of dyslipidemia underscore the persistent metabolic risks in PCOS, a relationship that was well documented in two studies, one by Mosorin et al. and the other by Goodman et al., which linked insulin resistance, hyperandrogenism, and persistent acne [47,48].

PCOS promotes acne through two primary pathways: androgen excess and insulin resistance. Elevated androgens stimulate sebaceous gland hypertrophy, increase sebum production, and promote follicular hyper-keratinization. Studies indicated that even mild biochemical hyperandrogenism has been shown to correlate with increased acne lesion counts and inflammatory activity. In a case–control study, Cappel et al. demonstrated that serum levels of dehydroepiandrosterone sulfate (DHEAS), dihydrotestosterone (DHT), and insulin-like growth factor 1 (IGF-1) were significantly associated with acne severity in adult women, supporting the role of androgenic activity in the pathogenesis of acne vulgaris [49]. Additionally, adult female acne frequently serves as one of the earliest clinical indicators of an underlying endocrine imbalance, and guidelines suggest that PCOS should be investigated routinely in such patients. As highlighted in a clinical review by Carmina et al., adult female acne is considered as a possible clinical expression of hyperandrogenism [50], and many PCOS guidelines recommend evaluating women with persistent acne for signs of androgen excess, hirsutism, menstrual irregularities, and metabolic disturbances.

From a diagnostic perspective, the Rotterdam criteria remain the most widely adopted for PCOS diagnosis, requiring any two of the following: oligo/anovulation, clinical or biochemical hyperandrogenism, and polycystic ovarian morphology. Our patient met these criteria, confirming the endocrine origin of her acne [51]. Insulin resistance, a core metabolic derangement in PCOS, further contributes to acne by suppressing hepatic SHBG synthesis, thereby increasing free testosterone, and by amplifying IGF-1, mediating follicular inflammation. In a prospective cohort of women with PCOS and acne, Kartal et al. demonstrated that higher HOMA-IR values were significantly linked to more severe acne presentations [52].

The topical regimen prescribed in this case, which included benzoyl peroxide, azelaic acid, and adapalene, was carefully selected to address multiple acne pathways. Research conducted by Thielitz et al. and Zaenglein et al. indicated that adapalene, a third-generation retinoid, promoted comedolysis and dermal remodeling, and its efficacy in patients with early atrophic scarring was well-supported [43,53]. Alternating it with topical antibiotics on days of lower tolerance enhanced adherence and reduced the risk of irritation and resistance. Adjunctive procedures also played a valuable role. The patient underwent monthly microneedling sessions, which likely accelerated dermal remodeling and improved the appearance of atrophic scars. This is in line with studies by El-Domyati et al. and Asif et al., who demonstrated that microneedling significantly improved post-acne scarring and collagen deposition, especially when paired with platelet-rich plasma (PRP) [54,55]. Moreover, a larger randomized controlled trial by Fabbrocini et al., involving 12 patients, reported that microneedling followed by PRP produced superior improvement in Goodman and Baron’s scar scale compared to microneedling alone (*p* < 0.05) [56]. Complementing these findings, a 2021 meta-analysis by Kang and Lu, who reviewed 14 controlled studies, including both RCTs and non-RCTs, found that combining microneedling with PRP yielded significantly greater clinical improvement, collagen deposition, and patient satisfaction, without increasing adverse events [57].

In summary, this case highlights the importance of a multimodal, interdisciplinary approach in treating PCOS-related acne. The integration of hormonal regulation, anti-inflammatory antibiotics, topical agents, and procedural support proved effective in the short term. However, as demonstrated by the relapse after COC discontinuation, long-term control requires continued metabolic monitoring and, when necessary, the addition of insulin-sensitizing agents, such as metformin, or the adoption of lifestyle and dietary interventions.

The therapeutic approach in Case 3 illustrates an evidence-based, multidisciplinary strategy for adult-onset acne with underlying metabolic syndrome, with systemic doxycycline selected for its proven efficacy in adult female acne without overt hyperandrogenism [58]. In contrast to Case 2, hormonal therapy was avoided due to the absence of hyperandrogenism and the presence of metabolic risk factors. This decision is in line with the current results of Branisteanu et al. and Dreno et al., who emphasize individualized hormonal evaluation in adult women with acne and caution against indiscriminate hormonal treatment in patients with dyslipidemia or insulin resistance [59,60].

The introduction of metformin by the diabetologist addressed a core pathogenic component, insulin resistance, a key driver of sebaceous hyperactivity and comedogenesis. Metformin improves insulin sensitivity, reduces circulating insulin and IGF-1, and increases SHBG, thereby mitigating androgen-driven acne mechanisms, as detailed by Albalat et al. and Kamboj et al. [61,62]. This mechanism of action is strongly supported by multiple clinical studies. Nguyen et al. conducted a systematic review of 15 controlled trials comprising over 1000 participants (primarily women with PCOS) and found that metformin led to significant reductions in acne severity in 13 studies, with 7 achieving statistical significance [63]. Similarly, a robust meta-analysis by Yen et al. systematically reviewed 51 clinical trials involving 2405 women with PCOS-related acne. The authors found that metformin, used alone or as adjunctive therapy, was associated with a significant reduction in acne severity and nearly halved the odds of persistent acne [64]. Furthermore, studies highlighted that metformin therapy reduces free bioactive IGF-I levels and restores insulin and testosterone balance [65], and a review, conducted by Andreadi et al., confirmed that metformin suppresses IGF-1 signaling, increases IGF-binding protein-3 (IGFBP-3), and helps rebalance gut microbiota, collectively dampening sebocyte proliferation [66]. Together, these data provide compelling evidence that targeting insulin resistance with metformin is a mechanistically justified and clinically effective adjunct strategy in acne management.

In our case, metformin not only contributed to dermatologic improvement but also helped normalize fasting glucose, reduce weight, and prevent the need for hormonal therapy, aligning with findings reported by Shamin et al. and Szefler et al. [67,68]. A recent trial by Sadati et al. emphasized that metformin offered comparable acne resolution to oral antibiotics while also improving metabolic markers [69].

Topical therapy in this case, comprising adapalene, benzoyl peroxide, azelaic acid, and occasional topical antibiotics, aligned with global acne treatment guidelines and was tailored to the patient’s tolerance and lesion type. The fixed combination of adapalene 0.1% and benzoyl peroxide 2.5% has demonstrated superior efficacy in improving acne lesion counts and maintaining good tolerability compared to monotherapy, particularly in adult-onset acne. In a large randomized, double-blind, controlled trial, Thiboutot et al. evaluated this combination in 517 patients and reported significantly greater reductions in both inflammatory and non-inflammatory lesion counts as early as the first week of treatment, with continued improvement through week 12. The fixed-dose combination outperformed either adapalene or benzoyl peroxide alone, while maintaining a favorable safety profile with only mild, transient cutaneous irritation in a minority of patients. These findings support its use as a first-line option in adult women, in whom skin sensitivity and persistent inflammatory lesions are common features [70].

Azelaic acid has been shown to be non-inferior to clindamycin for treating mild-to-moderate acne, with added benefits like reducing post-inflammatory hyperpigmentation and maintaining safety during pregnancy. In multicenter, randomized trials, 15% azelaic acid gel achieved comparable median reductions in inflammatory lesions (70–71%) versus clindamycin 1%, while demonstrating a superior tolerability profile—factors particularly advantageous in adult female patients [71].

Adjunctive monthly comedone extraction, while often underutilized, played a valuable role in expediting the removal of retention lesions, particularly relevant in adult women with slower epidermal turnover and persistent seborrhea [72]. This is consistent with the findings of Wise and Graber, who demonstrated that combining comedone extraction with classical therapy led to significantly better outcomes in patients with macrocomedones [73].

This case reinforces the notion that acne in adults, particularly late-onset forms, may be an early dermatologic signal of systemic imbalance, including metabolic syndrome. The integration of metabolic evaluation and treatment into acne management protocols is not only rational but necessary for sustained remission and relapse prevention. Collaborative care between dermatologists, endocrinologists, and primary care physicians is vital to identifying such cases early and optimizing both skin and systemic health outcomes.

Across all cases, oral doxycycline was selected for its dual antibacterial and anti-inflammatory properties, leading to significant reductions in lesion count and erythema. Its role as a first-line systemic agent in moderate-to-severe inflammatory acne is well established in international guidelines, particularly when topical therapy alone is insufficient [74,75]. Combination with retinoids or benzoyl peroxide further enhanced outcomes and limited resistance development [43]. Antioxidant-based skincare was used to counteract oxidative stress, especially relevant in metabolically driven acne. Monthly cosmetic procedures supported lesion clearance and dermal remodeling, contributing to scar prevention. This multifaceted, personalized approach reflects current treatment standards, which prioritize combination therapy, hormonal or metabolic targeting when indicated, and adaptation to underlying etiology [25]. The consistently favorable responses reinforce the importance of multidisciplinary care. Coordination among dermatologists, endocrinologists, and other specialists ensures comprehensive management, while psychological support remains essential for patients facing emotional distress from chronic acne.

However, this study is subject to the inherent limitations of case series design. The small sample size restricts the generalization of findings, and the absence of a control group limits the ability to draw causal conclusions. Laboratory and imaging parameters were tailored to each case rather than standardized across the series, which may introduce variability in interpretation. Additionally, psychological and quality-of-life measures, while clinically acknowledged, were not systematically assessed. Nonetheless, the observations presented are hypothesis-generating and highlight the importance of individualized care pathways in acne management, particularly when systemic comorbidities are involved.

Despite these limitations, this case series provides valuable clinical insight into the management of treatment-resistant or atypical acne vulgaris in the context of systemic comorbidities. A key strength lies in its multidisciplinary approach, which integrates dermatologic, endocrinologic, gynecologic, and metabolic perspectives, emphasizing the need to view acne as a potential marker of underlying systemic dysfunction. The detailed documentation of therapeutic response, relapse, and long-term management strategies enhances its practical relevance for clinicians facing similar complex cases.

## 5. Conclusions

The three cases illustrate how persistent acne can reflect diverse systemic contributors, ranging from corticosteroid misuse to endocrine or metabolic dysregulation. Successful outcomes were achieved through individualized treatment guided by a thorough diagnostic workup and multidisciplinary care. This reinforces the view of acne not merely as a cutaneous condition but as a possible clinical expression of underlying systemic imbalance. For adult female patients with atypical or resistant acne, early etiological reassessment and tailored interventions may be essential to achieve long-term therapeutic control. These cases may also serve as practical prompts for clinicians to consider broader endocrine and metabolic screening in patients whose acne does not follow classical patterns or fails to respond to standard therapy.

## Figures and Tables

**Table 1:** Therapeutic protocol for Case 1: corticosteroid-induced acneiform eruption.

Therapeutic Component	Product/Approach
Systemic antibiotics	Doxycycline 100 mg/day, for 3 months
Antihistamine	Desloratadine 5 mg/day, for 3 weeks
Corticosteroid withdrawal	Abrupt discontinuation of methylprednisolone aceponate 0.1%
Cleanser	Soap-free gel with zinc gluconate and niacinamide, supplemented by micellar water
Barrier-repair moisturizer	Ceramide-based emollient with panthenol, applied twice daily
Topical anti-acne therapy	Benzoyl peroxide 5% (morning), azelaic acid 15–20% (evening), clindamycin/erythromycin (alternated)
Photoprotection	SPF 50+, non-comedogenic fluid, applied daily
Cosmetic procedures	Monthly manual comedone extraction under sterile conditions
Maintenance phase	Cleansing + retinoid- or fruit acid-based creams 2–3×/week for long-term prevention

**Table 2:** Therapeutic protocol for Case 2: persistent acne associated with PCOS.

Therapeutic Component	Product/Approach
Systemic antibiotics	Doxycycline 100 mg/day, for 3 months
Hormonal therapy	Combined oral contraceptive (ethinylestradiol + cyproterone acetate), for 6 months
Cleanser	Gentle gel cleanser, soap-free, for acne-prone skin (twice daily)
Morning topical therapy	Benzoyl peroxide 5% (alternating with azelaic acid 20%)
Evening topical therapy	Adapalene 0.1% (first choice) or clindamycin/erythromycin on inflamed lesions
Moisturizer	Non-comedogenic cream with hyaluronic acid, used after actives
Photoprotection	SPF 50+, non-comedogenic, broad spectrum, applied daily
Procedures	Monthly comedone extraction and microneedling (dermapen) for atrophic scarring
Maintenance strategy	Extended retinoid-based evening routine; monitoring of metabolic profile

**Table 3:** Therapeutic protocol for Case 3: adult-onset acne with metabolic syndrome.

Therapeutic Component	Product/Approach
Systemic antibiotics	Doxycycline 100 mg/day, for 3–4 months
Topical regimen	AM: benzoyl peroxide 5% or azelaic acid 20%; PM: adapalene 0.1% or clindamycin/erythromycin
Cleanser	Soap-free, non-irritating cleanser for acne-prone skin (2×/day)
Moisturizer	Non-comedogenic cream with niacinamide, as needed
Photoprotection	Broad-spectrum SPF 50+, non-comedogenic formulation
Lifestyle changes	Hypocaloric Mediterranean-style diet + aerobic exercise (150 min/week)
Metabolic therapy	Metformin 500–1000 mg/day initiated by diabetologist
Cosmetic procedures	Monthly comedone extraction to assist lesion clearance
Maintenance phase	Ongoing topical retinoids and cosmetic follow-up, adapted to response and tolerance

**Table 4:** Summary of clinical presentation, treatment, and outcomes in three female patients with refractory acne.

Parameter	Case 1	Case 2	Case 3
Age (years)	21	26	39
Acne type	Steroid-induced acne	Moderate-to-severe acne (PCOS-related)	Adult-onset acne (metabolic syndrome)
Comedones	Present	Numerous	Present
Inflammatory lesions	Papules, pustules	Papules, pustules, nodules	Moderate papules
Atrophic scarring	Punctate atrophic scars	Present	Absent
Seborrhea	Marked	Marked	Moderate
Baseline GEA grade	3	4	3
Systemic treatment	Doxycycline 100 mg/day, antihistamines	Doxycycline 100 mg/day+ combined oral contraceptives	Doxycycline + metformin
Topical treatment	Benzoyl peroxide, azelaic acid, topical antibiotics	Adapalene, benzoyl peroxide, azelaic acid	Tretinoin, benzoyl peroxide, niacinamide
Cosmetic procedures	Manual comedone extraction	Comedone extraction, microneedling	Monthly comedone extraction
Response at intermediary evaluation	Gradual improvement/GEA grade 3	Significant improvement/GEA grade 2	Modest reduction GEA grade 3
Response at 6 months	Near-complete remission/GEA grade 1	Near remission/GEA grade 1	Partial remission/GEA grade 2

## Data Availability

Data are available from the last author upon request.

## Abbreviations

The following abbreviations are used in this manuscript:

BMI	Body mass index
*C. acnes*	*Cutibacterium acnes*
COC	Combined oral contraceptive
GEA	Global Evaluation Acne
IGF-1	Insulin-like growth factor 1
IL	Interleukin
LDL	Low-density lipoprotein
LTB_4_	Leukotriene B4
mg	Milligram
NF-κB	Nuclear factor kappa B
PCOS	Polycystic ovary syndrome
TLR	Toll-like receptors
